# Paediatric Longitudinal Tracheal Laceration From Blunt Force Trauma: A Case Report

**DOI:** 10.7759/cureus.19867

**Published:** 2021-11-24

**Authors:** Anna Loroch, John F Curran, David M Wynne

**Affiliations:** 1 ENT, NHS Greater Glasgow and Clyde, Glasgow, GBR; 2 Department of ENT Surgery, Queen Elizabeth University Hospital, Glasgow, GBR; 3 Paediatric Otolaryngology, Royal Hospital for Sick Children, Glasgow, GBR

**Keywords:** paediatric trauma, airway disruption, paediatric blunt trauma, tracheal laceration, tracheobronchial injury

## Abstract

Tracheal lacerations in the paediatric population are not common; however, they can be life-threatening. Prompt diagnosis and management are essential for a good prognosis.

Here, we present the case of a nine-year-old boy who presented to the hospital following a bicycle handlebar injury with neck pain and subcutaneous emphysema of the anterior thorax and neck. Chest X-ray revealed pneumomediastinum and a small pneumothorax. A computed tomography scan revealed a posterior longitudinal laceration of the trachea, measuring 1.5 cm, located superior to the carina at T1/2. As the patient was clinically stable, did not require any supplemental oxygen, and the tear was smaller than 2 cm, conservative management with steroids and broad-spectrum antibiotics was implemented. The patient was transferred to a tertiary ENT centre in Glasgow for observation in the paediatric intensive care unit where he recovered uneventfully. A repeat cross-sectional imaging six days after the injury revealed successful healing of the laceration.

Non-surgical management of a tracheobronchial injury can be an effective approach. This can be considered in the case of tears measuring <2 cm and in clinically stable patients. Imaging-based diagnosis in the case of patients with minor injuries who are improving with conservative treatment may be sufficient, and confirmation with bronchoscopy would be of questionable clinical value in such patients.

## Introduction

Tracheal lacerations are serious and life-threatening injuries that require immediate emergency care and often surgical intervention. These injuries are uncommon (with tracheobronchial injuries [TBIs] making up only 0.2-0.8% of trauma cases reaching the emergency department), especially in the paediatric population, and most of them are fatal at the scene of the incident [1].

Clinical presentation depends on the degree of laceration. In case of small tears, dry cough and subcutaneous emphysema typically follow, whereas sizeable lacerations can lead to respiratory distress with rapidly spreading emphysema and cyanosis, as well as haemoptysis [2].

TBIs may be traumatic (in children more commonly with a blunt force) or iatrogenic (following traumatic intubation/tracheostomy tube insertion, foreign body removal, etc.). Spontaneous tracheal rupture following severe coughing has also been reported [3].

Tears are presumed to arise due to expansion of the U-shaped tracheal cartilage secondary to a sudden increase in pressure in the tracheobronchial tree [1,4,5]. Most tears are located within 2-3 cm from the carina and the right bronchus is more predominantly affected due to its fixation in the thorax [1,2].

Prompt diagnosis and management of TBI are crucial for a patient’s prognosis, which can be especially challenging in polytrauma cases [5,6].

## Case presentation

A nine-year-old boy presented to Ninewells Hospital in Dundee immediately following blunt trauma to the chest, resulting in the sudden appearance of subcutaneous emphysema. The patient reported falling on his bicycle’s handlebars, hurting his chest, with the impact landing superior to his left sternoclavicular joint. He denied sustaining a head injury or losing consciousness. He returned home right after the incident, having noted increasing neck swelling and reporting neck pain. The patient’s mother indicated that she noticed a difference in her son’s voice, describing it as more high-pitched.

**Figure 1:**
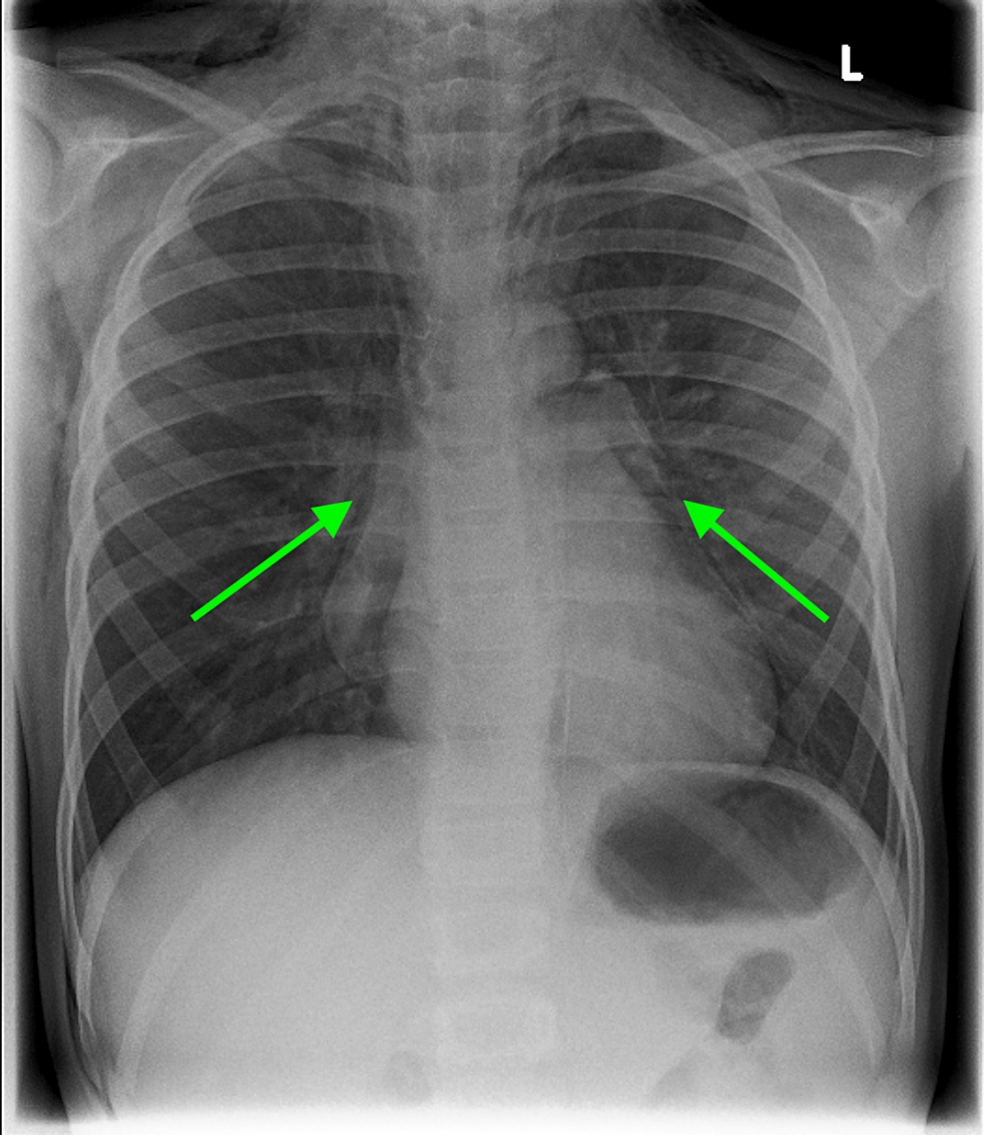
Chest X-ray (anteroposterior view) revealing marked subcutaneous emphysema and pneumomediastinum (angel wing sign, green arrows) with a small left-sided pneumothorax.

He was clinically well on admission and stated that oedema and pain started subsiding by the time of the assessment. He had normal work of breathing and did not require any oxygen therapy. Examination revealed emphysema bilaterally of the anterior thorax and neck all the way up to the left eye, as well as a small area of erythema on the anterior chest wall. Chest X-ray (CXR) performed in the emergency department revealed marked subcutaneous emphysema and pneumomediastinum with small left-sided pneumothorax, but no evidence of rib fractures (Figure 1).

**Figure 2:**
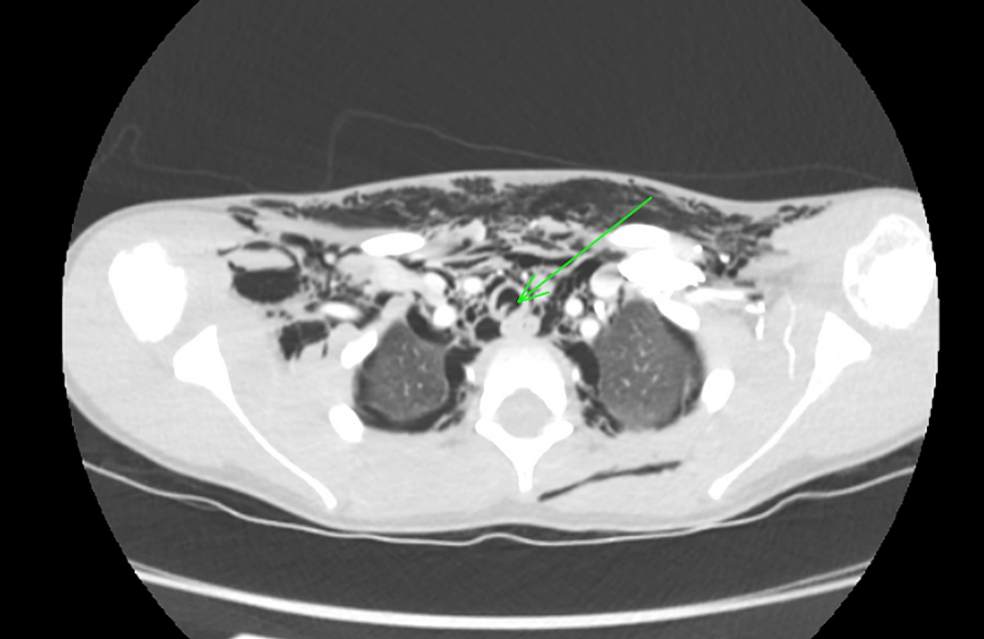
CT of the neck and thorax (axial plane) revealing a 1.5 cm tear of the trachea at the level of T1-2; a linear defect running craniocaudally was noted in the posterior wall of the trachea approximately 4 cm above the carina (green arrow). CT: computed tomography

Trauma computed tomography (CT) imaging followed, revealing a large pneumomediastinum, bilateral thin pneumothoraces, as well as a 1.5 cm tear of the trachea at the level of T1-2. A linear defect running craniocaudally was noted in the posterior wall of the trachea approximately 4 cm above the carina (Figure 2). Flexible nasendoscopy was performed shortly after the scan, visualising the nasopharynx, oropharynx, and larynx; the only abnormality noted was oedema of the posterior pharyngeal wall.

A decision was made to undertake conservative management (with low threshold for intervention and securing airway with intubation and cuff inflated below the tracheal injury if needed), commence steroids and intravenous antibiotics, and position the head at 30 degrees. Two doses of intravenous dexamethasone (4.9 mg) were administered prior.

The patient was discussed with the tertiary paediatric ENT centre, Royal Hospital for Children (RHC), in Glasgow, and he was subsequently transferred to the Paediatric Intensive Care Unit at RHC for observation. Two doses of intravenous dexamethasone (4.9 mg) were administered prior to transfer and a broad-spectrum antibiotic (co-amoxiclav) was commenced.

He spent two days in intensive care and did not require ventilatory support or supplemental oxygen. Surgical emphysema and neck oedema were slowly decreasing and the patient had an uneventful recovery. The steroid course was stopped once he was deemed fit for ward-level care, and the antibiotics were continued (intravenously) until discharge (seven days in total).

**Figure 3:**
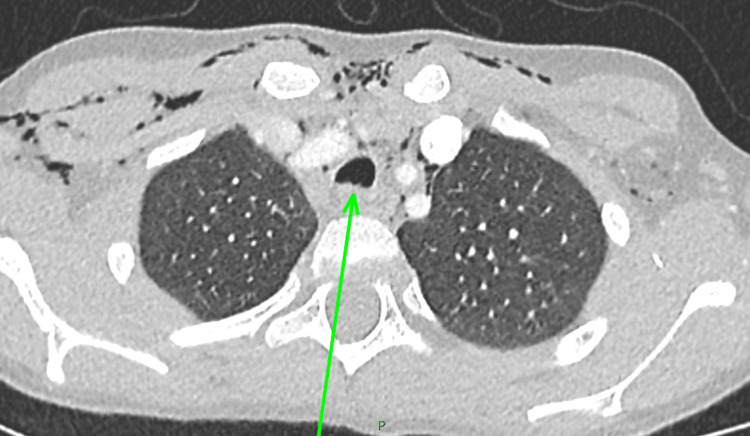
A follow-up CT of the neck and thorax (axial plane) revealing a healing tracheal laceration (green arrow). CT: computed tomography

Cross-sectional imaging of the neck and thorax was repeated six days after the initial scan, revealing persistent irregularity of the posterior tracheal wall, but no identifiable tear and residual subcutaneous emphysema, which was significantly reduced in volume (Figure 3). The patient was then deemed fit for discharge.

## Discussion

TBI in the paediatric population is caused by blunt trauma in 94% of cases [1]. Our patient sustained the laceration following a bike accident. Handlebar injuries in children are common and often do not bear significant consequences. They are, however, deemed to have a high potential to crush the visceral organs; hence, a meticulous clinical examination should be performed and doctors should have a low threshold for imaging (as it could help exclude potential insidious damage) [7].

The pattern of injuries can be explained by anatomical differences in paediatric patients. Given the elasticity of the thoracic cavity, lack or paucity of external damage does not exclude underlying internal organ injuries (as in our patient’s case, only mild erythema of the anterior thorax was noted) [5].

Strong clinical suspicion of TBI can be reached based on clinical examination, especially in the presence of large lacerations, which will likely cause respiratory distress with cyanosis, rapidly increasing subcutaneous and mediastinal emphysema, and severe cough [1,4]. Advanced trauma life support management should be initiated for all trauma patients as it was proven to significantly decrease mortality for patients with TBIs [1].

Imaging can be a useful tool to confirm the diagnosis. CXR, although not diagnostic for the site of the lesion, can indicate the presence of emphysema, the air beneath the cervical fascia, pneumothorax, or atelectasis. A fallen lung sign is pathognomonic of a total rupture in the main bronchi [5,8].

The use of CT in children remains controversial; however, it has a high sensitivity for tracheal rupture detection [3]. In our patient’s case, it served as a primary diagnostic tool, indicating the site and size of the tracheal laceration, along with its complications.

Bronchoscopy/fiberoptic endoscopy has been the gold standard for definitive diagnosis of TBIs and can serve as a useful tool not only to allow locating the injury but also provide adequate ventilation by adjusting the position of the endotracheal tube or even undertaking immediate management of minor tears with tissue adhesives [6].

As our patient was clinically stable, did not require supplemental oxygen or mechanical ventilation throughout his hospitalisation, and a small tear was visualised with cross-sectional imaging, it was deemed unnecessary to proceed with endoscopy as it would not alter the management at the time. Gati et al. noted that in some cases bronchoscopy failed to confirm the diagnosis of small TBIs found on a CT scan and suggested that, even though endoscopy is recommended for early diagnosis, close observation following an imaging-based diagnosis can be considered based on clinical assessment [4,6]. It is important to provide care in the appropriate environment, that is, tertiary paediatric centre, high dependency unit, or intensive care unit, depending on the patient’s clinical condition. If a transfer needs to be arranged, it is crucial to bear in mind potential complications that can arise and ensure an appropriate level of care is available during the commute.

Surgical management used to be a mainstay treatment of tracheal lacerations. However, a recent review of contemporary management strategies of blunt TBIs advocates that a conservative approach in certain cases can be an equally effective alternative [1]. Jougon et al. also suggested considering a non-surgical approach for delayed diagnosis or late presentation. Their proposed treatment included broad-spectrum antibiotics and anti-inflammatory aerosols [9,10], while our patient received a course of broad-spectrum antibiotics and steroids (intravenous dexamethasone). Small tears requiring intubation could be managed with a cuff being placed distal to the site of the injury or selective bronchial intubation [3,6,11]. The use of extracorporeal membrane oxygenation was also reported as a means to provide cardiorespiratory support in paediatric patients who sustained blunt trauma to the chest [1,5]. The purpose of surgical management is to ensure sufficient airway patency, repair the damage causing the air leak, and prevent complications of spontaneous healing [1].

Using Cardilo’s classification for endoscopic assessment of injuries can be a useful guide regarding the treatment strategy. Surgical intervention is advised for clinically unstable patients with tears larger than 2 cm [1,4,6].

Follow-ups for patients with TBIs should be arranged. The literature suggests repeat bronchoscopy [8,12]; however, as the diagnosis in our patient was based on CT imaging, a repeat scan was arranged which confirmed healing of the tracheal tear, resolution of the pneumothoraces and pneumomediastinum, and improvement in the appearances of the emphysema.

## Conclusions

Because TBIs are rare but potentially fatal, it is important to quickly establish the diagnosis and commence appropriate management. It is crucial to employ the ATLS protocol in trauma cases and to follow the primary and secondary surveys. If there is suspicion of TBI, particularly if patients present with rapidly increasing subcutaneous emphysema, respiratory distress, cough, and neck pain, a multidisciplinary team approach is suggested and early involvement of the otolaryngology team is strongly advised.

Bronchoscopy is recommended as the gold standard investigation for definitive diagnosis. However, we would argue that in certain cases, especially a clinically stable patient and a small (<2 cm) tracheal laceration, imaging-based diagnosis may suffice to establish the diagnosis and decide on the management strategy. If non-surgical treatment is decided upon, close monitoring in an intensive care unit is advisable.
